# Charge Separating Microfiltration Membrane with pH-Dependent Selectivity

**DOI:** 10.3390/polym11010003

**Published:** 2018-12-20

**Authors:** Daniel Breite, Marco Went, Andrea Prager, Mathias Kühnert, Agnes Schulze

**Affiliations:** Leibniz Institute of Surface Engineering (IOM), Permoserstr. 15, 04318 Leipzig, Germany; daniel.breite@iom-leipzig.de (D.B.); marco.went@iom-leipzig.de (M.W.); andrea.prager@iom-leipzig.de (A.P.); mathias.kuehnert@iom-leipzig.de (M.K.)

**Keywords:** polymer membrane, charge selective, microfiltration, surface modification, zwitterionic

## Abstract

Membrane filters are designed for selective separation of components from a mixture. While separation by size might be the most common approach, other characteristics like charge can also be used for separation as presented in this study. Here, a polyether sulfone membrane was modified to create a zwitterionic surface. Depending on the pH value of the surrounding solution the membrane surface will be either negatively or positively charged. Thus, the charged state can be easily adjusted even by small changes of the pH value of the solution. Charged polystyrene beads were used as model reagent to investigate the pH dependent selectivity of the membrane. It was found that electrostatic forces are dominating the interactions between polystyrene beads and membrane surface during the filtration. This enables a complete control of the membrane’s selectivity according to the electrostatic interactions. Furthermore, differently charged beads marked with fluorescent dyes were used to investigate the selectivity of mixtures of charged components. These different components were successfully separated according to their charged state proving the selectivity of the invented membrane.

## 1. Introduction

The main purpose of a polymer membrane is the specific separation of components from e.g., a solution. The success of this separation process is defined by the membrane selectivity, which depends on the characteristics of the components to be separated. Accordingly, selectivity can be achieved due to e.g., the component’s size, affinity, or charge. So far, charge or ion selective membranes are used in the field of nanofiltration and reverse osmosis, where charged ions are retained, and therefore removed from the solution. A recent example was presented by Ge et al. where acids were recovered from a complex feed stream while other unwanted ions were removed [1]. Furthermore, ion selective membranes are utilized for electrodialysis [2] or in case of catalytic membranes [3].

Nevertheless, the separation based on the charge of the components is also interesting for microfiltration applications. So far, only a few studies can be found in literature. Emin et al., for example, presented the pH-controlled separation of bovine serum albumin and hemoglobin based on the membrane surface charge [4]. Other examples of charge selective microfiltration membranes are the polyelectrolyte-filled membranes studied by Mika et al. [5] or the sulfonated PVDF membranes prepared by Baroña and coworkers [6].

Previous studies also revealed that electrostatic interactions dominate the fouling tendencies of polymer membranes [7,8,9]. If membrane and foulants are oppositely charged electrostatic attraction will occur leading to adsorption of the foulant and consequently to heavy membrane fouling. On the contrary, if membrane and foulant are evenly charged electrostatic repulsion prevents the adsorption of foulants. Therefore, the here presented work advanced previous studies with the focus on the development of a charge selective microfiltration membrane.

The investigation of interactions between membrane surface and dissolved components is of great importance as they influence e.g., the membrane selectivity or the fouling tendencies, or both [10,11]. Especially membrane fouling was detailed investigated. Most of the time, fouling was explained by hydrophobic interactions [12,13,14,15,16,17,18,19,20]. Thus, many membrane modification approaches focused on hydrophilization by grafting [13,15,16,18,20,21,22], electron-beam irradiation [23,24,25], or plasma treatment [11,14,26,27,28,29,30,31]. Recently, more emphasis is placed on the role of electrostatic interactions in membrane science [32,33,34,35,36,37,38,39,40]. Consequently, more insight into the possible interactions between membrane and dissolved components like e.g., proteins or particles is gained. 

The Derjaguin–Landau–Verwey–Overbeek [41,42] theory and its extended version by van Oss [43,44] describe the different types of interactions that need to be considered regarding two interacting surfaces. Here, electrostatic interactions, hydrophobic interactions, hydrogen bonds, or acid–base interactions are taken into consideration to calculate an overall interaction between two surfaces. As both surfaces are in contact with an electrolyte, solution parameters like ionic strength or pH value are also of importance. This is especially true for electrostatic interactions, which are related to the pH dependent surface potentials [32,37,39]. In this study, we want to make use of this dependency by creation of a zwitterionic surface structure. This structure can switch its charged state from positive to negative charges depending on the pH value of the surrounding solution. 

Furthermore, next to the membrane surface also the characteristics of the interacting components from the solution need to be considered. Examples for differently charged components are proteins of comparable size. Unfortunately, there are no two proteins which differ only in charge, but size, conformation, and shape change as well. To exclude such issues in comparability, polystyrene (PS) beads will be used in this study. They are spherical, cannot be deformed, and their size and charge can be tuned according to the synthesis parameters [45,46]. Therefore, PS beads are ideal model components to investigate the separation selectivity of a zwitterionic membrane. Furthermore, PS beads are also available with different fluorescent markers, which enables an optical discrimination of differently charged (and labeled) PS beads filtered as a mixture. 

## 2. Materials and Methods

### 2.1. Reagents and Materials

Polyether sulfone (PES) flat sheet membranes were prepared by non-solvent induced phase separation (NIPS). Chemicals were purchased from Sigma Aldrich (St. Louis, MO, USA): aluminum oxide (Brockmann activity I, Fluka), 2,2′-azobis(2-methylpropionamidine) dihydrochloride (AIBA), lauryldimethylammonia acetate, latex beads (~1 µm, amine-modified, fluorescent red, L2778), latex beads (~1 µm, carboxylate-modified, fluorescent yellow-green, L4655), lysine, 1-methyl-2-pyrrolidone (NMP), potassium persulfate (KPS), styrene. The 2-Aminoethyl methacrylate hydrochloride (AEMA, Acros Organics), polyethylene glycol (PEG, 400 g mol^−1^, Acros Organics), and sodium bicarbonate were purchased from Thermo Fisher Scientific (Geel, Belgium). Other purchased chemicals: glutaraldehyde (GA, Merck, Darmstadt, Germany), *n*-hexane (Merck, Darmstadt, Germany), hydrochloric acid solution (0.1 M, VWR, Radnor, DE, USA), polyether sulfone (PES, Ultrason E2010, BASF, Ludwigshafen, Germany), sodium carbonate (anhydrate, VWR, Radnor, DE, USA), sodium hydroxide solution (0.1 M, VWR, Radnor, DE, USA). 

All chemicals were used as received. Ultrapure water was taken from a MilliQ-System (Merck Millipore, Billerica, MA, USA). The dialysis membranes used for the bead purification were obtained from Carl Roth (cellulose acetate, Nadir, molecular weight cut-off (MWCO): 10–20 kDa, Wiesbaden, Germany).

### 2.2. Membrane Preparation

PES membranes were prepared by NIPS. A polymer solution (14 wt.% PES, 65 wt.% PEG, and 21 wt.% NMP) was casted (200 µm gap, ZWA 2121 Wasag Applicator, Zehntner Testing Instruments, Sissach, Switzerland) on a glass plate and kept in humidified air for 5 min. Precipitation was performed in a water bath (~10 °C, 10 min), followed by washing the membranes with pure water three times for 30 min, respectively.

### 2.3. Membrane Modification

PES membranes were modified by electron beam (E-Beam) irradiation using a self-made electron accelerator in nitrogen atmosphere (O_2_ quantities <10 ppm). Membrane samples were immersed into an aqueous solution of AEMA (2 wt.%) and irradiated using a dose of 200 kGy [7,47]. The membranes were washed with pure water three times for 30 min, respectively. To create the zwitterionic membrane surface the membranes were then immersed into a GA solution (2 wt.%, pH 9.2 (carbonate/bicarbonate buffer), 2 h), roughly rinsed with water, and subsequently immersed into a lysine solution (2 wt.%, pH 9.2 (carbonate/bicarbonate buffer), 2 h). Finally, all membrane samples were washed three times for 30 min, respectively, and stored in water until usage.

### 2.4. Membrane Characterization

PES membrane samples were investigated before and after modification with zwitterionic moieties. Scanning electron microscopy (SEM), measurement of water permeance, and mercury porosimetry were used to investigate the membrane morphology. X-ray photoelectron spectroscopy (XPS), measurement of zeta potential, and water contact angle (WCA) measurements were carried out to analyze the chemical composition of the membrane surfaces.

SEM (Ultra 55 SEM, Carl Zeiss Ltd., Goettingen, Germany) was used to investigate the membrane morphology. A chromium coating (30 nm, Z400 sputter system, Leybold, Hanau, Germany) was used to prevent charging of the samples.

Water permeance was calculated based on filtration experiments using a stainless steel pressure filter holder (16249, Sartorius, Goettingen, Germany) for dead end filtration. An amount of 100 mL of deionized water were filtered through the membrane (active area: 17.3 cm^2^) at 1 bar, and the time of flow-through was recorded. Values of at least three different (independently prepared) samples were averaged. Pure water permeance *J* was calculated following Equation (1).

(1) $$J=\frac{V}{t\cdot A\cdot p}$$

*V* is the volume of water, *t* is the time of flow-through, *A* is the active area, and *p* is the applied pressure.

Porosity and pore size distribution were measured with a mercury porosimeter (PoreMaster 30, Quantachrome Instruments, Odelzhausen, Germany). Values of at least three different samples were averaged.

X-ray photoelectron spectroscopy (XPS, Kratos Axis Ultra, Kratos Analytical Ltd., Manchester, UK) was used to analyze the chemical composition of the membrane surface. Values of at least three different (independently prepared) samples were averaged.

Surface wettability was measured using a static water contact angle measurements system (DSA 30E, Krüss, Hamburg, Germany) and the sessile drop method. Values of at least three different (independently prepared) samples were averaged.

Zeta potentials were measured using streaming potential measurements performed with the adjustable gap cell in the SurPASS system (Anton Paar, Graz, Austria). The zeta potential *ζ* was calculated based on the Smoluchowski Equation (2). Values of at least three different (independently prepared) samples were averaged.

(2) $$\zeta =\frac{dU}{dp}\cdot \frac{\eta }{\varepsilon \cdot \varepsilon_{0}}\cdot \kappa$$

*U* is the streaming potential, *p* is the pressure, *η* is the viscosity of the electrolyte solution, *ε* is the dielectric constant of the electrolyte solution, *ε*_0_ is the vacuum permittivity and *κ* is the electrolyte conductivity.

The stability of the modified membranes was investigated via Soxhlet extraction using water as solvent. Membrane samples were extracted for 5 d at 100 °C followed by membrane characterization (permeance, Hg porosimetry, water contact angle, SEM, XPS, zeta potential) as described above.

### 2.5. Preparation and Characteriztion of PS Beads

PS beads were prepared by an emulsion polymerization method as described in our previous study. The initiator used for the polymerization reaction determined the surface charge of the PS particles. KPS [48] was used to generate negatively charged PS beads, while AIBA [49] resulted in positively charged particles. Measurement of the particle’s size was carried out using the Zetasizer System from Malvern (Zetasizer Nano ZS with multipurpose titrator MPT-2, Malvern Instruments, Worcestershire, UK) as well as SEM. The Zetasizer system was also used to measure the zeta potential of the PS beads.

### 2.6. Filtration of PS Beads

Filtration experiments with PS beads (~200 nm) were carried out via dead-end filtration using a 50 mL stirred cell (Amicon, Merck Millipore, Billerica, MA, USA) and the respective 1 L reservoir. The membrane was mounted in the stirred cell and 140 mL of water (pH value adjusted to pH 4 with HCl or pH 9 with NaOH) were passed through the membrane to measure pure water permeance as well as to adjust the membrane to the desired pH value. The permeance was calculated based on the time of flow-through as described in Section 2.4. Afterwards, 150 mL of a solution of PS beads (~10 mg L^−1^, pH value adjusted as described above) was passed through the membrane. The permeate was collected in portions of 25 mL and the permeance was calculated per 25 mL portion. The PS bead concentration of the feed solution and of the different permeate portions was determined spectrometrically (Infinite M200, Tecan, Mannedorf, Switzerland) using the UV absorption of styrene at 290 nm.

### 2.7. Static Binding of PS Beads

Static adsorption experiments were conducted using the fluorescent PS beads (~1 µm) described in Section 2.1 (filtration of those larger particles was not possible due to the particles being larger than the membrane pore size). Samples of the PES-Lysine membrane (1 cm discs) were placed in a 48-well microtiter plate and an aqueous solution (pH value adjusted to pH 4 with HCl or pH 9 with NaOH) was added to adjust the membrane samples to the respective pH value. The samples were shaken for 5 min, followed by removing the aqueous solutions and replacing them with PS bead solutions of the respective pH value. High diluted (2.5 ppm) solutions of either aminated or carboxylated PS beads were used, and the samples were shaken overnight, followed by washing the samples with water three times for 30 min, respectively.

The binding of the PS beads was investigated by fluorescent microscopy (Olympus IX71, light source: X-Cite Series 120 PC Q, software: Cellsens, Olympus, Tokyo, Japan). To simultaneously excite both types of PS beads a blue light source (494 nm) was used resulting in a yellow fluorescence of the aminated (positively charged) PS beads and a green fluorescence of the carboxylated (negatively charged) PS beads.

## 3. Results and Discussion

The aim of this study was to develop a membrane with adaptable surface charge based on the pH value of the surrounding solution. This enables a selective filtration of charged components from a mixture. The following Section 3.1 will briefly describe the physico-chemical properties of the PES-Lysine membrane, while the selective filtration/adsorption experiments will be presented in the Section 3.2 and Section 3.3.

### 3.1. Zwitterionic PES–Lysine Membrane

**Figure 1:**
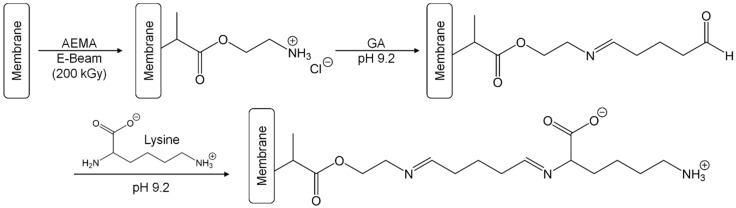
The synthesis of the PES–Lysine membrane is a three-step process starting with the E-Beam-induced grafting-from reaction with AEMA. Subsequently, a Schiff base reaction with GA is performed at pH 9.2, followed by another Schiff base reaction with the α-amino group of lysine at pH 9.2.

The zwitterionic PES–Lysine membrane is based on a self-made PES microfiltration membrane. This membrane was first functionalized via an E-Beam-induced grafting-from reaction with AEMA (Figure 1). While the membrane is irradiated with electrons in wet state, a number of different activated species is formed on the membrane surface leading to initiation of a radical polymerization of the acrylate monomers on the membrane surface. Subsequent chain growth reactions lead to the formation of brush-like polymers containing amino groups. These amino groups were transformed to a Schiff base with GA at pH 9.2. Finally, the remaining aldehyde group of GA reacts with the α-amino group (pKa 8.9 [50]) of the amino acid lysine. The second amino group (pKa 10.3 [50]) is protonated at the used pH value, and is therefore not involved in the reaction. The membrane is equipped with a negatively charged carboxylic acid group (pKa 2.2 [50]) and a positively charged amino group (pKa 10.3). Unlike other lysine-based modification like e.g., the immobilization of poly(l-lysine) [51], the membrane modification reported here results in a zwitterionic membrane surface.

**Figure 2:**
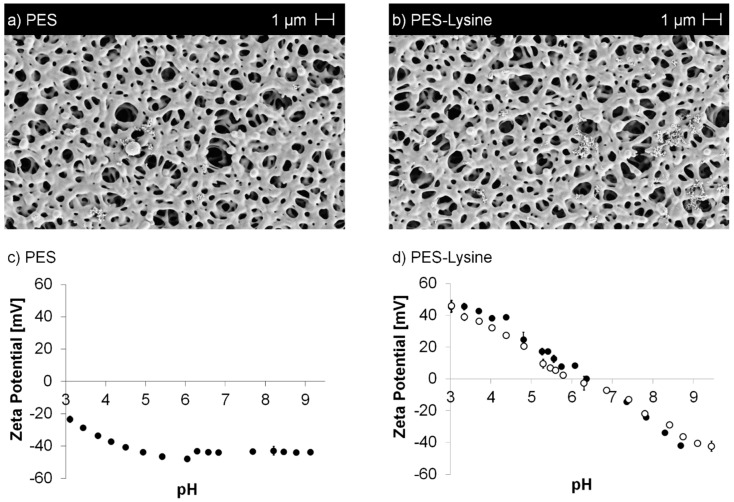
SEM images (**a**,**b**) of the pristine PES and PES–Lysine membrane as well as zeta potential vs. pH curves (**c**,**d**) of the pristine PES and PES–Lysine (before (full symbol) and after (open symbol) stability test) membrane.

A detailed characterization of the membranes was conducted and the results are summarized in Table 1 (all error values represent standard deviations). In comparison to the pristine membrane, no significant changes in water permeance, porosity, and average pore size were observed. SEM images (Figure 2a,b) confirm no impact on the membrane structure avoiding also pore blocking by the applied modification reactions.

However, the chemical properties of the membrane surface were significantly changed. The water contact angle was reduced by ~50° switching to a hydrophilic surface from the former hydrophobic pristine membrane. The surface properties were confirmed after the stability test (5 d Soxhlet extraction, as described in Section 2.4).

The successful modification was also confirmed by XPS, as nitrogen can be detected on the PES–Lysine membrane. The nitrogen can be attributed to the lysine moiety as well as to the AEMA functionalization applied in the first reaction. No changes were observed after the stability test. Furthermore, zeta potentials were determined according to streaming potential measurements (Figure 2c,d, error bars represent standard deviations). The pristine membrane shows the expected trend of zeta potential vs. pH value, which is usually observed for unmodified polymeric materials [52,53]. Due the hydroxide ions (originating from the surrounding solution) adsorbed to the polymer surface [52,53], the surface occurs to have a negative zeta potential over a broad range of pH. In addition, sulfonic acid groups might be present within the PES polymer leading to an even more acid characteristic. The isoelectric point (IEP) was determined at a pH value of ~2. In comparison, the PES-Lysine membrane has an IEP in the neutral range at a pH value of 6.2. This value is close to the value, which could be theoretically expected (arithmetic average of the pKa values) given the pKa values of the present carboxylic acid and amino functionalities. The zeta potential varies from positive values at low pH to negative values at high pH. This is the result of the immobilized zwitterionic moieties, which contain carboxylic acid groups as well as amino groups. At low pH the carboxylic acid groups are protonated and therefore uncharged, while the amino groups have a positive charge in their protonated state, resulting in an overall positive charge. Contrary, amino groups are deprotonated and uncharged at high pH, while the carboxylic acid groups become negatively charged in their deprotonated state, resulting in an overall negative charge. No significant changes were observed after the stability test.

In summary, the development of a zwitterionic PES-Lysine membrane was successful. The applied modification strategy resulted in a very thin layer of the lysine moiety on top of the PES membrane. Thus, the morphology (porosity, pore size) was not changed. The presence of the covalently immobilized zwitterionic lysine enables the self-adaption of the charged state of the membrane surface according to the pH value of the surrounding solution.

### 3.2. Selective Filtration

**Figure 3:**
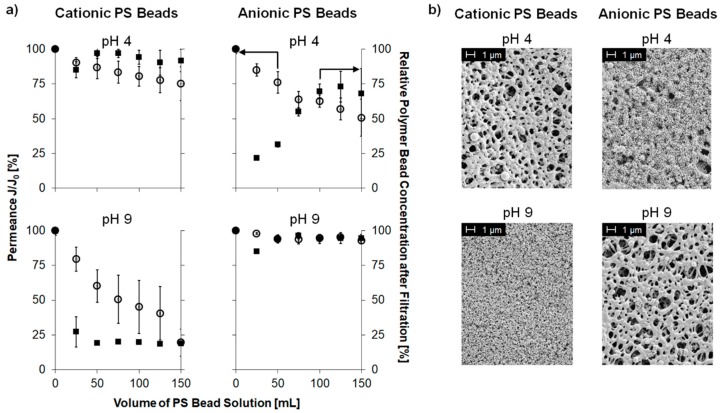
(**a**) Permeance *J/J*_0_ (open circles, left axis) and relative PS bead concentration in the filtrate (full squares, right axis) of a polystyrene (PS) bead solution (cationic or anionic) filtered through a PES–Lysine membrane at pH value of 4 or 9, respectively; (**b**) SEM images of the membrane’s top side after filtration.

The selective filtration of charged PS beads through the PES–Lysine membrane in a dead-end filtration approach was investigated. The permeance and the concentration of PS beads were monitored for every 25 mL of permeate (Figure 3a, all error bars represent standard deviations). Furthermore, SEM images were taken from the membrane’s top side after filtration (Figure 3b).

Filtration experiments carried out at pH 4 with positively charged PS beads resulted in a slight decline (25%) in permeance while the concentration of PS beads in the permeate did not change. In addition, no pore blocking was observed in the corresponding SEM images. It can be concluded, that the (at pH 4) positively charged membrane repelled the positively charged PS beads due to electrostatic repulsion. Thus, the particles passed the membrane because of them being smaller than the average pore diameter of the membrane, and no pore blocking occurred.

On the contrary, cationic PS beads adsorbed to the membrane surface due to electrostatic attraction. Here, permeance (~50% reduction) as well as PS bead concentration (~25% reduction) declined. Interestingly, the concentration of PS beads recovered after ~50 mL of PS bead solution. Here, a first layer of positively charged PS beads was formed on the membrane (as can be seen in the respective SEM image), which compensates the negative charge of the membrane. Thus, the overall surface charge of the membrane is again positive resulting in electrostatic repulsion of the following PS beads, which can then pass the membrane. This is an important finding as it shows the limits of this type of membrane. Once the surface charge is compensated by oppositely charged components of the solution, the desired selectivity might be lost.

Filtration experiments carried out at pH 9 gave opposite results because the membrane surface is negatively charged compared to a positive surface charge at pH 4. No decline in permeance or in PS beads concentration in the permeate were observed in case of anionic PS beads due to the electrostatic repulsion. Accordingly, no adsorbed PS beads were observed in the respective SEM images.

On the other hand, filtration experiments with negatively charged beads at pH 4 resulted in instantaneous decline of permeance (~75% reduction) and nearly no remaining particles (~75% reduction) in the permeate. Nevertheless, this filtration experiment results in a completely blocked membrane structure as observed by SEM. This can be explained by electrostatic attraction between the positively charged membrane and the negatively charged PS beads. A recovery of the concentration of PS beads as described for anionic beads at pH 4 was not observed in this case, because the membrane was already completely blocked by cationic PS beads when the surface charge of the membrane was compensated. Thus, no more PS beads could pass through the membrane.

### 3.3. Selective Adsorption

The filtration experiments described above demonstrated that the zwitterionic membrane can switch selectivity by adjusting the pH value to let either positively or negatively charged particles pass the membrane. For visualization of the membrane’s selectivity also in case of mixtures of differently charged particles, PS beads labelled with different fluorescent dyes were used. Anionic PS beads can be detected by green fluorescence; cationic PS beads gave a yellow fluorescence, so they could be identified by fluorescence microcopy. The labelled PS beads were too large for filtration experiments (Particle size of ~1 µm compared to ~200 nm particles in the filtration experiments, average pore size of the PES membranes: 0.6 µm). Therefore, adsorption experiments were carried out instead.

**Figure 4:**
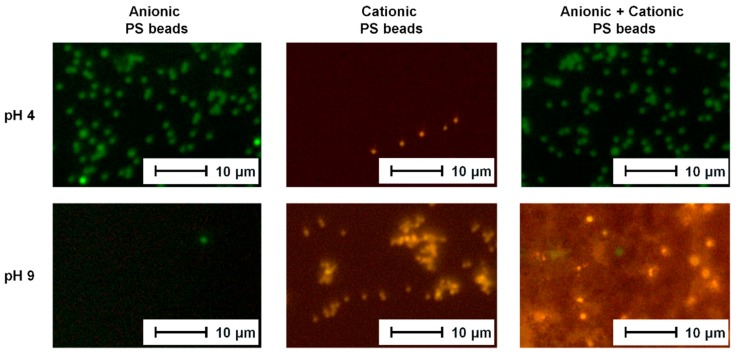
Fluorescence microscopy images of PS beads (anionic/cationic/mixture) adsorbed to the PES–Lysine membrane at pH 4 and pH 9, respectively.

The resulting fluorescence microscopy images are presented in Figure 4. The adsorption experiments carried out with using just one type of PS beads confirmed the filtration results discussed in Section 3.2. Anionic PS beads (green) adsorbed to the positively charged PES–Lysine membrane at pH 4, and cationic PS beads (yellow) adsorbed at pH 9 when the membrane was negatively charged. In case of evenly charged membrane surface and PS beads no adsorption occurred and no fluorescent PS beads can be observed in the respective images.

New insight was gained from the adsorption experiments using a mixture of positively and negatively charged PS beads. The charged PS beads were mixed at high dilution to ensure that no precipitation of the PS beads occurs due to the electrostatic attraction of the oppositely charged PS beads. The experiment carried out at pH 4 (positively charged membrane surface) resulted in the adsorption of green (anionic) PS beads as a result of electrostatic attraction. The cationic PS beads (yellow) did not adsorb because of electrostatic repulsion. Fluorescent microscopy images of experiments carried out at pH 9 (negatively charged membrane surface) show predominantly yellow (cationic) PS beads. Only a small number of green (anionic) PS were detected. Again, oppositely charged PS beads were attracted while evenly charged once were repelled. Thus, it can be concluded that the selectively already observed in the filtration experiments was confirmed also by adsorption experiments. Furthermore, the developed membrane can be used to separate mixtures of differently charged components, too.

## 4. Conclusions

The development of a zwitterionic PES–Lysine membrane using E-Beam technology was successfully accomplished. The covalently immobilized lysine resulted in a charge selective microfiltration membrane. The aim of this study was to investigate the self-adaption of the membrane surface according to the pH value of the surrounding medium. Filtration and adsorption experiments were carried out using oppositely charged PS beads. It was revealed that:

- Electrostatic interaction are important during both filtration and adsorption processes. In case of evenly charged surfaces, no adsorption occurs due to electrostatic repulsion, while electrostatic attraction results in adsorption due to electrostatic attraction.
- Mixtures of differently charged PS beads can be selectively separated by switching the pH value of the solution, and therefore, the respective charged state of the PES–Lysine membrane. While evenly charged components can pass the membrane, oppositely charged PS beads are attracted to the membrane.
- Retention of PS beads (during filtration) was ~80% for cationic PS beads at pH 9 and ~30% for anionic PS beads at pH 4 (after high adsorption during initial phase). Filtration of cationic beads at pH 4 and anionic beads at pH 9 resulted in no significant retention.
- Once the membrane surface is completely covered by a layer of the oppositely charged components, the selectivity of this membrane is lost or reversed. Thus, the adsorption capacity of such a membrane needs to be considered.

It can be concluded that the zwitterionic membrane presented in this study could also be applied in separation of mixtures of real samples (i.e., proteins). The pH value needs to be adjusted to achieve the selectivity described above, but the needed pH values (pH 4 or 9) are not jet critical to many proteins and denaturation can be avoided. Thus, the invented membrane modification could also be applied in various separation applications.

The approach chosen for this study used PS beads as model components and filtration experiments were carried out in dead-end mode. This way, it was possible to prove the feasibility of the designed membrane. Nevertheless, further factors (e.g., ionic strength, buffer solutions, etc.) might need to be considered for real applications like the separation of proteins, which will be the focus of future studies.

## Figures and Tables

**Table 1:** Characteristics of pristine PES and PES–Lysine membrane.

Membrane	Permeance	Porosity	Average Pore Size	Water Contact Angle	Chemical Composition (XPS) [rel. atom-%]			
	[L (m² h bar)^−1^]	[%]	[μm]	[°]	C	O	S	N
PES	14,200 ± 2000	72 ± 2	0.65 ± 0.04	102 ± 4	75 ± 1	20 ± 2	5 ± 1	-
PES-Lysine	11,700 ± 500	76 ± 1	0.62 ± 0.01	54 ± 7	74 ± 2	20 ± 2	4 ± 1	2 ± 1
PES-Lysine *	10,000 ± 500	71 ± 4	0.64 ± 0.04	48 ± 8	73 ± 2	20 ± 1	5 ± 1	2 ± 1

* Membrane characterization after stability testing (5 d Soxhlet extraction in water at 100 °C).
